# Association of chronic non-cancer pain status and buprenorphine treatment retention among individuals with opioid use disorder: Results from electronic health record data

**DOI:** 10.1016/j.dadr.2022.100048

**Published:** 2022-03-26

**Authors:** William S. John, Paolo Mannelli, Rick H. Hoyle, Lawrence Greenblatt, Li-Tzy Wu

**Affiliations:** a Department of Psychiatry and Behavioral Sciences, Division of Social and Community Psychiatry, Duke University Medical Center, Durham, NC, United States; b Department of Pyschiatry and Behavioral Sciences, Division of Adult Psychiatry and Psychology, Duke University Medical Center, Durham, NC, United States; c Department of Psychology and Neuroscience, Duke University, Durham, NC, United States; d Department of Medicine, Division of General Internal Medicine, Duke University Medical Center, Durham, NC, United States; e Duke Institue for Brain Sciences, Duke University, Durham, NC, United States; f Center for Child and Family Policy, Sanford School of Public Policy, Duke University, Durham, NC, United States

**Keywords:** Opioid use disorder, Buprenorphine, Chronic pain

## Abstract

**Background:**

Although chronic non-cancer pain (CNCP) is common among individuals with opioid use disorder (OUD), its impact on buprenorphine treatment retention is unclear. The goal of this study was to use electronic health record (EHR) data to examine the association of CNCP status and 6-month buprenorphine retention among patients with OUD.

**Methods:**

We analyzed EHR data of patients with OUD who received buprenorphine treatment in an academic healthcare system between 2010 and 2020 (*N* = 676). We used Kaplan-Meier curves and Cox proportional hazards regression to estimate risk of buprenorphine treatment discontinuation (≥90 days between subsequent prescriptions). We used Poisson regression to estimate the association of CNCP and the number of buprenorphine prescriptions over 6 months.

**Results:**

Compared to those without CNCP, a higher proportion of patients with CNCP were of older age and had comorbid diagnoses for psychiatric and substance use disorders. There were no differences in the probability of buprenorphine treatment continuation over 6 months by CNCP status (*p* = 0.15). In the adjusted cox regression model, the presence of CNCP was not associated with time to buprenorphine treatment discontinuation (HR = 0.90, *p* = 0.28). CNCP status was associated with a higher number of prescriptions over 6 months (IRR = 1.20, *p* < 0.01).

**Conclusions:**

These findings suggest that the presence of CNCP alone cannot be reliably associated with buprenorphine retention in patients with OUD. Nonetheless, providers should be aware of the association between CNCP and greater psychiatric comorbidity among patients with OUD when developing treatment plans. Research on the influence of additional characteristics of CNCP on treatment retention is needed.

## Highlights

• EHR data of patients receiving buprenorphine treatment for OUD was analyzed.
• 27.4% of patients with OUD receiving buprenorphine treatment had chronic pain.
• Chronic pain was associated with greater psychiatric comorbidity in our sample.
• Chronic pain status was not associated with buprenorphine treatment retention.

## 1 Introduction

Chronic non-cancer pain (CNCP), defined as persistent pain lasting for at least 3 months or longer (Merskey and Bogduk, 1994), is highly prevalent among individuals with opioid use disorder (OUD). Research in medical settings show that up to approximately two-thirds of patients with OUD have a CNCP condition (Hser et al., 2017; John and Wu, 2020; Voon et al., 2017). Moreover, national survey data indicate that approximately two-thirds of individuals who misused opioids in the past-year reported physical pain relief to be their primary reason for opioid misuse (Han et al., 2017).

Opioid agonist treatment (OAT) using buprenorphine is a widely used, efficacious, and relatively accessible approach for managing OUD symptoms (i.e., craving and withdrawal) and facilitating recovery (Mattick et al., 2014). However, research suggests that co-occurring CNCP among individuals with OUD may be a prognostic factor for adverse outcomes during buprenorphine treatment (Ditre et al., 2019). Due to a lack of established guidelines for treating co-occurring OUD and CNCP, treatment for OUD may often be prioritized, which could result in leaving pain symptoms undertreated (Berg et al., 2009). This, in turn, poses a risk for relapse or other substance use to manage pain symptoms (i.e., self-medication). Indeed, some studies among patients with OUD have shown that the presence of chronic pain is associated with increased craving during opioid agonist treatment using buprenorphine or methadone (Tsui et al., 2016), and increased odds of opioid and other substance use following detoxification (Larson et al., 2007; Potter et al., 2010). It has also been shown that greater pain persistence and pain volatility during buprenorphine treatment is associated with increased odds of opioid use and frequency of use during treatment (Worley et al., 2015, 2017). Other studies, however, have shown no association between chronic pain and continued opioid use in patients receiving opioid agonist treatment (Dhingra et al., 2015; Ilgen et al., 2006; Potter et al., 2015). Furthermore, untreated chronic pain often results in disability or mental health comorbidity such as depression or anxiety, which in turn may negatively affect aspects of psychosocial functioning and quality of life essential to OUD recovery (Speed et al., 2018).

The association between buprenorphine treatment and improved clinical outcomes is particularly dependent upon the duration of treatment. Studies show that longer periods of buprenorphine treatment retention with greater continuity are associated with lower rates of mortality, overdose, relapse, and costly healthcare utilization (Fiellin et al., 2008; Hser et al., 2016; Kornor et al., 2007; Lo-Ciganic et al., 2016; Manhapra et al., 2018; Parran et al., 2010; Schwarz et al., 2012; Sordo et al., 2017). Yet, buprenorphine retention rates are relatively low among individuals with OUD, with most studies showing that only about half of patients remain in treatment at 6 months since initiation (Fiellin et al., 2008; Lo-Ciganic et al., 2016; Manhapra et al., 2018; Schwarz et al., 2012; Timko et al., 2016). The extent to which co-occurring CNCP among patients with OUD affects buprenorphine treatment retention is not fully understood. In fact, previous research has largely shown no association between CNCP and buprenorphine treatment retention among patients with OUD (Bounes et al., 2013; Fox et al., 2012; Peck et al., 2021), despite reports that chronic/persistent pain negatively affects other treatment outcomes such as craving and relapse to opioid use (Larson et al., 2007; Potter et al., 2010; Tsui et al., 2016). However, firm conclusions from previous research on the association of CNCP and treatment retention are limited by small sample sizes or restrictive inclusion/exclusion criteria from clinical trial samples, and little consideration of factors that may interact with CNCP to influence effects on retention.

The objective of the present study was to further investigate the impact of CNCP status on buprenorphine treatment retention. The main component of this study extending previous work was the leveraging of electronic health record (EHR) data from a large academic healthcare system. The use of EHR data is advantageous given its capability to provide patients’ prescription and diagnosis information and real-world data that can be generalizable to patients receiving OUD care in the community. This study also extended previous work by examining the association of CNCP and retention by type of chronic pain condition as well as the interaction of CNCP and other key factors known to influence retention including age, sex, race/ethnicity, comorbid psychiatric disorders, and buprenorphine dose.

## 2 Methods

### 2.1 Data source

We used a retrospective study design, analyzing existing EHR data from the Duke University Health System (DUHS). The DUHS serves as the primary healthcare system for Durham County, North Carolina, which includes 3 hospitals and over 300 ambulatory clinics. The DUHS had nearly 2.2 million outpatient visits and over 66,000 inpatient stays in fiscal year 2020 (DUHS, 2021). All EHR data generated within the DUHS are stored within the Duke Medicine Enterprise Data Warehouse, which employs a formal extract, transform, and load procedure to integrate data from source systems on a nightly basis to ensure consistency and quality and to minimize redundancy (Horvath et al., 2014). The use of these data for analysis was approved by the DUHS Institutional Review Board.

### 2.2 Study sample

**Fig. 1:**
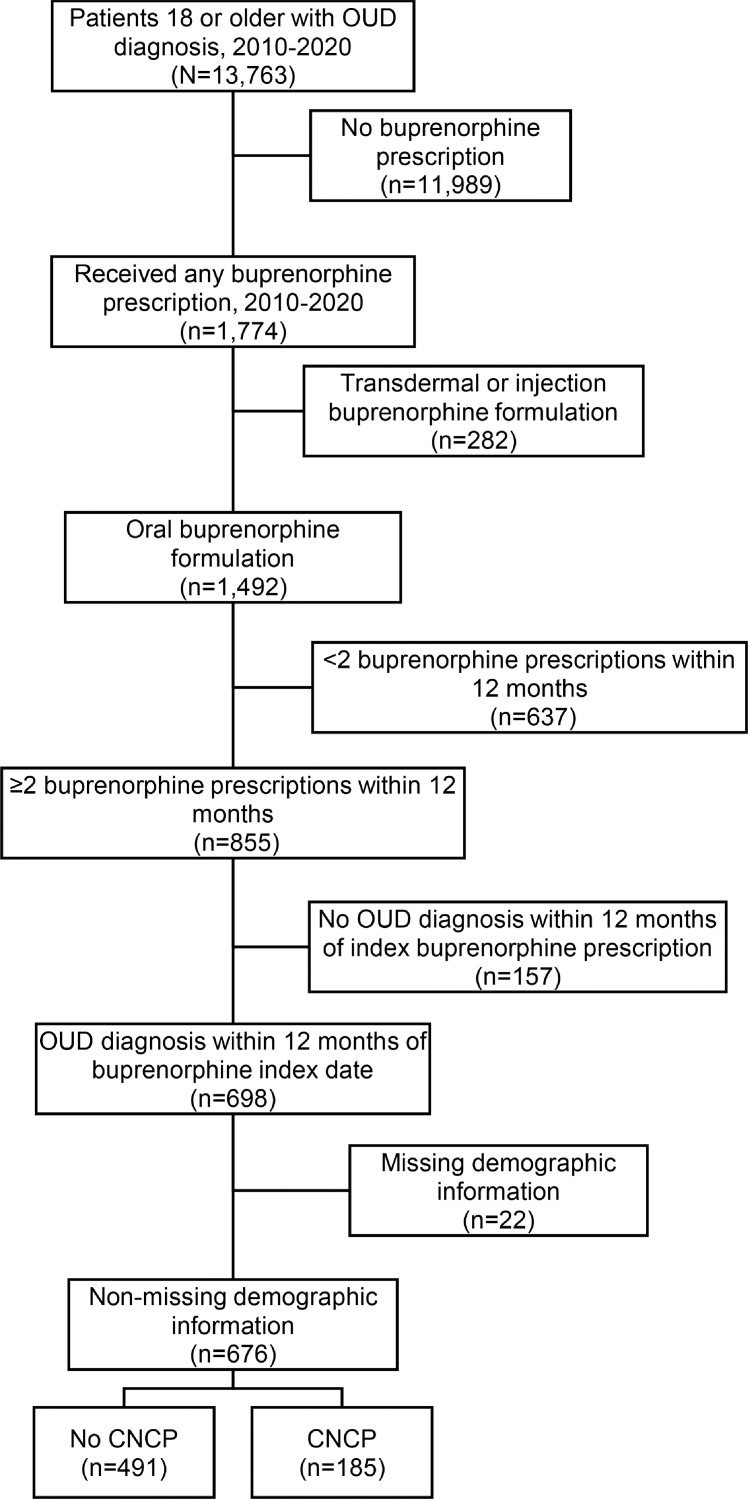
. Sample selection.

We analyzed EHR data from adult patients who received healthcare from a hospital or clinic within the DUHS between January 1, 2010 and December 31, 2020. Patients included in the study sample had an OUD diagnosis based on ICD-9/10-CM codes for opioid abuse or dependence and were aged 18 years or older as of January 1, 2010. We included patients who had at least two buprenorphine prescriptions during a 12-month period to increase the likelihood of only including patients receiving buprenorphine for maintenance treatment as opposed to detoxification (Lo-Ciganic et al., 2016). To increase the likelihood that patients included in the sample had buprenorphine prescribed for OUD and not pain, we excluded prescriptions for intravenous and transdermal formulations of buprenorphine and those without an OUD diagnosis within 12 months of the index buprenorphine prescription date. We also excluded any patients with missing demographic information. The creation of the study cohort is illustrated in Fig. 1.

### 2.3 Measures

#### 2.3.1 Outcome variables

The primary outcome was number of days to discontinuation of buprenorphine treatment within 6 months following the index buprenorphine prescription date. The index prescription was defined as the first prescription in the 12-month period of having ≥2 prescriptions. Discontinuation was defined as 90 days or more between subsequent buprenorphine prescriptions, as used in previous studies as well (Hui et al., 2017). As a secondary outcome, we examined the number of distinct buprenorphine prescriptions within 6 months following the index buprenorphine prescription date.

#### 2.3.2 Primary independent variable

Our primary independent variable of interest was the presence of CNCP. We used the International Classification of Diseases, 9⁠th revision, Clinical Modification (ICD-9-CM) and ICD-10-CM codes listed in the discharge or final diagnoses codes for outpatient, ED, or inpatient encounters to classify CNCP. We defined CNCP as having at least one ICD-9/10-CM code for any condition that may represent chronic pain and by not having an ICD-9/10-CM code for cancer-related pain (338.3, G89.3) within 6 months prior to the index buprenorphine prescription date (Table S1). The ICD-9/10-CM codes used to define CNCP were based on previous studies and those provided by the Centers for Disease Control and Prevention (CDC, 2018; Samples et al., 2018). We also assessed non-mutually exclusive categories for types of CNCP conditions that included general chronic pain, back pain, osteoarthrosis, musculoskeletal pain, and migraine/chronic headache.

#### 2.3.3 Covariates

Sociodemographic characteristics included sex, age, and patient-identified race/ethnicity categorized as non-Hispanic white, non-Hispanic Black/African-American, non-Hispanic other race (i.e., Asian, American Indian, Alaska Native, native Hawaiian or Pacific Islander, multiracial, or other), and Hispanic. We also assessed the baseline presence of psychiatric comorbidities within 6 months prior to the index buprenorphine prescription date. As with CNCP, psychiatric comorbidities were classified using ICD-9/10-CM codes listed in the discharge or final diagnoses codes for outpatient, ED, or inpatient encounters. Psychiatric comorbidities (excluding substance use disorders) included diagnostic codes for depression, any mood disorder (i.e., depressive, bipolar, or other mood-related disorder), anxiety, and other psychiatric disorder diagnoses (i.e., psychotic, sleep, adjustment, personality, attention-deficit/impulse/conduct, somatoform, or eating disorders) (Centers for Medicare and Medicaid Services CMS, 2019). Non-opioid substance use disorder comorbidities included diagnostic codes for tobacco, alcohol, stimulant (i.e., cocaine, amphetamine), and other drug (i.e., hallucinogen, inhalant, other/unspecified drug) use disorders. Furthermore, we included daily buprenorphine dose by measuring the daily dose in milligrams of the last prescription received prior to discontinuation or 6-month retention. Daily dose was categorized into low (<16 mg), medium (16–24 mg), or high (≥24 mg). We also included a category to capture those with missing dose data in order to minimize potential selection bias as a result of excluding these patients from the sample.

### 2.4 Data analysis

Descriptive statistics were used to examine the frequency of demographic variables, CNCP, and comorbid psychiatric disorders and SUDs among the overall sample and stratified by CNCP status. Differences in the demographic and clinical characteristics as a function of CNCP status were assessed using chi-square tests. Next, we generated Kaplan-Meier survival curves of buprenorphine discontinuation within 180 days by CNCP status. We used Cox (proportional hazards) regression models to estimate the association of CNCP status and time to buprenorphine treatment discontinuation. We used Poisson regression models to estimate the association of CNCP status and number of buprenorphine prescriptions. Covariates included patient demographic characteristics (sex, age, race/ethnicity), comorbid psychiatric disorders, comorbid SUDs, and daily dose of buprenorphine. To explore potential subgroup differences in the association of CNCP and our outcome measures, we used separate models that included an interaction term between CNCP and each covariate, while controlling for all other covariates.

## 3 Results

### 3.1 Sample characteristics

Among the overall sample of patients with OUD receiving buprenorphine treatment (*N* = 676), over half (53.3%) were male and over one-third (35.2%) were aged 35–49 years old (Table 1). Nearly two-thirds (63.9%) of the overall sample were white.

**Table 1:** Characteristics of patients with opioid use disorder receiving buprenorphine treatment by chronic non-cancer pain status.

	**OUD- overall**		**OUD without CNCP**		**OUD with CNCP**		
	*N* = 676		*N* = 491		*N* = 185		
**Characteristic**	N	Column% (SE)	N	Column% (SE)	N	Column% (SE)	*p*-value
**Sex**							
Male	360	53.3 (1.92)	255	51.9 (2.26)	105	56.8 (3.65)	0.26
Female	316	46.7 (1.92)	236	48.1 (2.26)	80	43.2 (3.65)	
**Age (years) on Jan. 1, 2010**							
18–25	185	27.4 (1.72)	146	29.7 (2.06)	39	21.1 (3)	<0.05
36–34	136	20.1 (1.54)	100	20.4 (1.82)	36	19.5 (2.91)	
35–49	238	35.2 (1.84)	171	34.8 (2.15)	67	36.2 (3.54)	
50+	117	17.3 (1.46)	74	15.1 (1.62)	43	23.2 (3.11)	
**Race/ethnicity**							
White, non-Hispanic	432	63.9 (1.85)	305	62.1 (2.19)	127	68.6 (3.41)	0.25
Black, non-Hispanic	214	31.7 (1.79)	164	33.4 (2.13)	50	27 (3.27)	
Other, Hispanic	30	4.4 (0.79)	22	4.5 (0.93)	8	4.3 (1.5)	
**Type of chronic pain condition**							
Any	185	27.4 (1.72)	0	0 (0)	185	100 (0)	n/a
General chronic pain	105	15.5 (1.39)	0	0 (0)	105	56.8 (3.65)	
Back pain	98	14.5 (1.36)	0	0 (0)	98	53 (3.67)	
Migraine/headache	13	1.9 (0.53)	0	0 (0)	13	7 (1.88)	
Osteoarthrosis	20	3 (0.65)	0	0 (0)	20	10.8 (2.28)	
Musculoskeletal pain	43	6.4 (0.94)	0	0 (0)	43	23.2 (3.11)	
**Comorbid psychiatric disorder diagnoses**							
Depressive disorder	78	11.5 (1.23)	28	5.7 (1.05)	50	27 (3.27)	<0.0001
Mood disorder (any)	110	16.3 (1.42)	40	8.1 (1.24)	70	37.8 (3.57)	<0.0001
Anxiety disorder (any)	97	14.3 (1.35)	38	7.7 (1.21)	59	31.9 (3.43)	<0.0001
Other psychiatric disorder§	71	10.5 (1.18)	20	4.1 (0.89)	51	27.6 (3.29)	<0.0001
Any psychiatric disorder	170	25.1 (1.67)	67	13.6 (1.55)	103	55.7 (3.66)	<0.0001
**Comorbid substance use disorders**							
Tobacco use disorder	85	12.6 (1.28)	33	6.7 (1.13)	52	28.1 (3.31)	<0.0001
Alcohol use disorder	44	6.5 (0.95)	22	4.5 (0.93)	22	11.9 (2.38)	<0.01
Stimulant use disorder	49	7.2 (1)	13	2.6 (0.73)	36	19.5 (2.91)	<0.0001
Other drug use disorder^¶^	73	10.8 (1.19)	31	6.3 (1.1)	42	22.7 (3.08)	<0.0001
**Daily dose of last buprenorphine prescription**							
Low (<16 mg)	344	50.9 (1.92)	255	51.9 (2.26)	89	48.1 (3.68)	0.21
Medium (16–23 mg)	211	31.2 (1.78)	150	30.5 (2.08)	61	33 (3.46)	
High (≥24 mg)	86	12.7 (1.28)	66	13.4 (1.54)	20	10.8 (2.28)	
Missing	35	5.2 (0.85)	20	4.1 (0.89)	15	8.1 (2.01)	

§ Other psychiatric disorders included psychotic, sleep, adjustment, personality, attention-deficit/impulse/conduct, somatoform, or eating disorders. ^¶^Other drug use disorders included cannabis, sedative/hypnotic/anxiolytic, hallucinogen, inhalant, or other/unspecified drug use disorder. OUD: opioid use disorder; SE: standard error; CNCP: chronic non-cancer pain.

Over one-fourth (*n* = 185; 27.4%) of the sample had a diagnosis for any CNCP condition within 6 months prior to the index buprenorphine prescription date. Regarding types of CNCP, diagnoses for general chronic pain were most prevalent in the sample (15.5%), followed by back pain (14.5%) and musculoskeletal pain (6.4%). Approximately one-fourth of the sample (25.1%) had a concurrent diagnosis for any psychiatric disorder (excluding SUD) diagnosis within 6 months of the index date, including 16.3% with any mood disorder diagnosis and 14.3% with any anxiety disorder diagnosis. Regarding comorbid substance use disorders, 12.6% of the sample had comorbid tobacco use disorder, 6.5% had alcohol use disorder, 7.2% had a stimulant use disorder, and 10.8% had a non-stimulant drug use disorder. About half of sample (50.9%) had a daily buprenorphine dose (i.e., according to last prescription prior to discontinuation or 6-month retention) less than 16 mg.

Compared to those without CNCP, a higher proportion of patients with CNCP were of older age and had concurrent diagnoses for a psychiatric disorder or substance use disorder (Table 1). Of note, over half (55.7%) of those with OUD and CNCP had a diagnosis for any psychiatric disorder compared to 13.6% of those without CNCP. There were no differences between groups in the proportion of patients by sex, race/ethnicity, or daily buprenorphine dose.

### 3.2 Time to buprenorphine treatment discontinuation

Approximately one-fourth of the sample (*n* = 173, 25.6%) discontinued buprenorphine treatment after the first prescription according to study criteria. By 10 days, half of the sample discontinued buprenorphine treatment (*n* = 337, 49.9%); by 32 days, an additional 16.7% discontinued treatment (*n* = 450, 66.6%); by 180 days, 96.9% of the sample (*n* = 655) discontinued treatment.

**Fig. 2:**
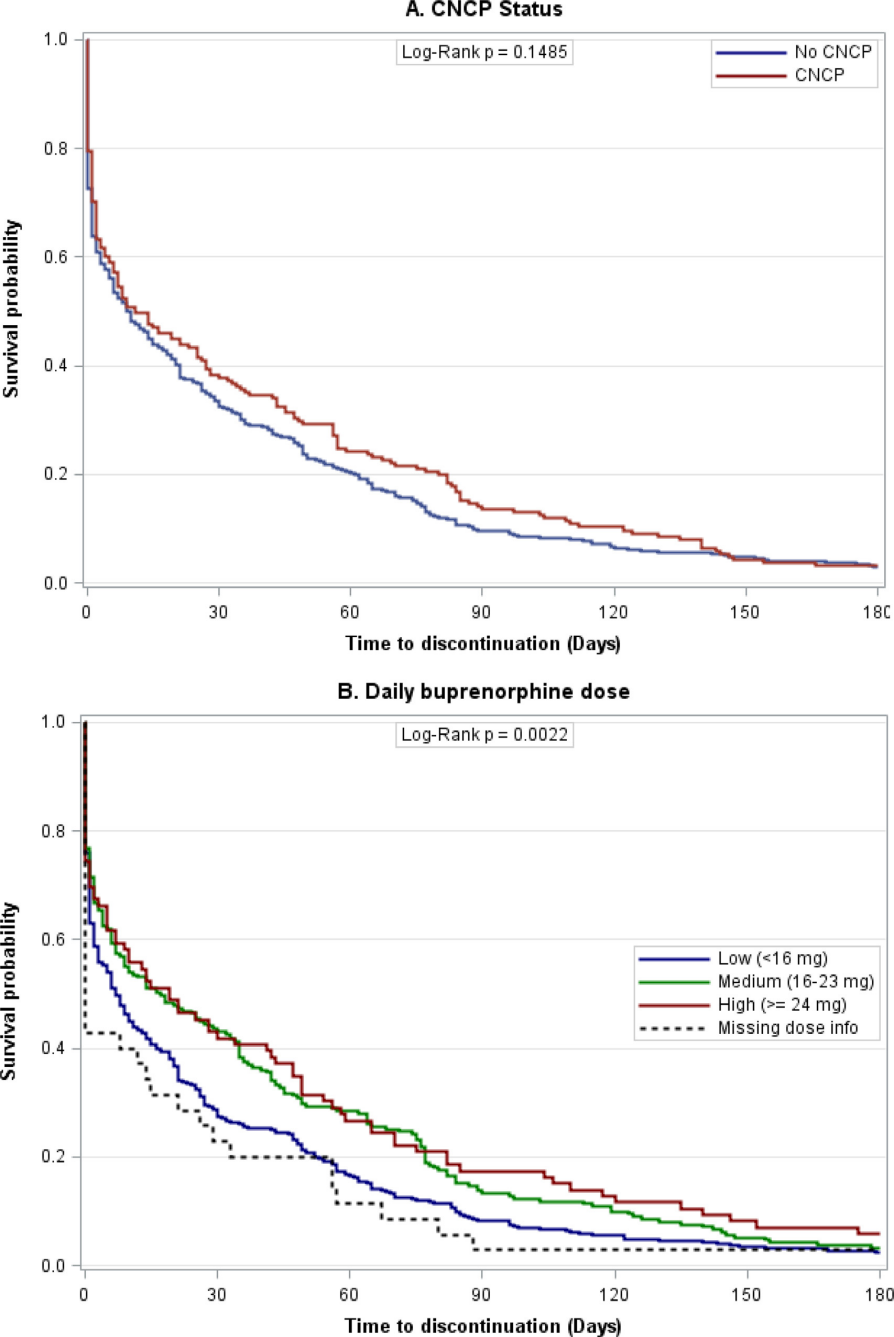
. Kaplan-Meier survival curves for probability of buprenorphine treatment discontinuation within 6 months of treatment initiation among patients with OUD.

As shown by Kaplan Meier survival curves, there was no difference in the probability of buprenorphine treatment continuation across 6 months by baseline CNCP status (*p* = 0.15; Fig. 2A). There was a significant difference in the probability of buprenorphine treatment continuation by buprenorphine dose (*p* < 0.05), which can be attributed to higher probability of discontinuation among those receiving lower (vs. higher) doses, respectively (Fig. 2B). In the adjusted Cox regression model, the presence of any CNCP condition at baseline was not associated with time to buprenorphine treatment discontinuation (Table 2). There was also no association of comorbid psychiatric disorders with time to treatment discontinuation. Compared to males, females had a lower hazard of discontinuation (HR = 0.82, *p* < 0.05). Comorbid substance use disorder was associated with a higher hazard of discontinuation (HR = 1.36, *p* < 0.05). Compared to lower daily buprenorphine doses (<16 mg/day), higher daily doses were associated with lower hazards of discontinuation (16–23 mg/day: HR = 0.77, *p* < 0.01; ≥24 mg/day: HR = 0.70, *p* < 0.01). In separate models, interaction effects between CNCP and each covariate were not found to be significant.

**Table 2:** Cox regression analysis of predictors of buprenorphine treatment discontinuation at 6 months.

	**Unadjusted model**			Adjusted model*		
**Variable**	**Hazard ratio**	**95% CI**	***p***-value	**Hazard ratio**	**95% CI**	***p***-value
**Sex**						
Male	Ref.	—-	—-	Ref.	—-	—-
Female	**0.84**	**0.72–0.99**	**< 0.05**	**0.82**	**0.69–0.96**	**<0.05**
**Age (years) on Jan. 1, 2010**						
18–25	Ref.	—-	—-	Ref.	—-	—-
36–34	0.99	0.79–1.24	0.96	1.03	0.82–1.29	0.81
35–49	0.91	0.75–1.10	0.34	0.86	0.70–1.06	0.15
50+	0.84	0.67–1.07	0.16	0.84	0.66–1.08	0.17
**Race/ethnicity**						
White, non-Hispanic				Ref.	—-	—-
Black, non-Hispanic	1.09	0.92–1.28	0.33	1.06	0.89–1.25	0.51
Other, Hispanic	0.87	0.59–1.28	0.48	0.85	0.58–1.26	0.43
**Type of CNCP condition**						
Any chronic pain	0.89	0.75–1.05	0.17	0.90	0.73–1.10	0.28
General chronic pain	**0.79**	**0.64–0.98**	**0.03**	—-	—-	—-
Back pain	0.88	0.71–1.10	0.27	—-	—-	—-
Migraine/headache	0.81	0.47–1.41	0.46	—-	—-	—-
Osteoarthrosis	0.81	0.51–1.28	0.36	—-	—-	—-
Musculoskeletal pain	0.96	0.7–1.31	0.78	—-	—-	—-
**Comorbid psychiatric disorder diagnoses**						
Any psychiatric disorder	0.88	0.74–1.05	0.17	0.85	0.69–1.06	0.15
Mood disorder (any)	0.82	0.66–1.01	0.06	—-	—-	—-
Anxiety disorder (any)	0.92	0.74–1.15	0.46	—-	—-	—-
Other psychiatric disorder^§^	0.92	0.71–1.18	0.49	—-	—-	—-
**Comorbid substance use disorders**						
Any substance use disorder	1.16	0.96–1.39	0.12	**1.27**	**1.04–1.55**	**<0.05**
Tobacco use disorder	1.10	0.87–1.38	0.44	—-	—-	—-
Alcohol use disorder	0.99	0.73–1.36	0.96	—-	—-	—-
Stimulant use disorder	1.00	0.75–1.35	0.98	—-	—-	—-
Other drug use disorder^¶^	1.27	1.00–1.63	0.05	—-	—-	—-
**Daily dose of last buprenorphine prescription**						
Low (<16 mg)	Ref.	—-	—-	Ref.	—-	—-
Medium (16–23 mg)	**0.80**	**0.67–0.95**	**< 0.01**	**0.70**	**0.55–0.90**	**<0.01**
High (≥24 mg)	**0.74**	**0.58–0.94**	**< 0.01**	**0.78**	**0.65–0.93**	**<0.01**
Missing	1.23	0.86–1.74	0.26	1.31	0.92–1.89	0.14

Note: Statistically significant (*p* < 0.05) results are bolded. *The adjusted model was controlled for the variables shown in column including sex, age, race/ethnicity, any chronic non-cancer pain, any comorbid psychiatric disorder, any comorbid substance use disorder, and daily buprenorphine dose. ^§^Other psychiatric disorders included psychotic, sleep, adjustment, personality, attention-deficit/impulse/conduct, somatoform, or eating disorders. ^¶^Other drug use disorders included cannabis, sedative/hypnotic/anxiolytic, hallucinogen, inhalant, or other/unspecified drug use disorder. CI: Confidence interval; CNCP: chronic non-cancer pain.

### 3.3 Number of buprenorphine prescriptions

Among the overall sample, the mean (SD) number of buprenorphine prescriptions received across 180 days was 2.76 (1.55). Patients with CNCP at baseline received a mean (SD) of 3.02 (1.63) prescriptions across 180 days; patients without CNCP received a mean (SD) of 2.67 (1.51) prescriptions. In the adjusted Poisson regression model, the presence of any CNCP diagnosis at baseline was associated with a higher number of prescriptions within 180 days of the index prescription (IRR = 1.20, *p* < 0.01) (Table 3). Additionally, receiving a medium daily buprenorphine dose (16–23 mg/day) was associated with a higher number of prescriptions compared to receiving a low daily dose (<16 mg/day) (IRR = 1.14, *p* < 0.05). In separate models, interaction effects between CNCP and each covariate were not significant.

**Table 3:** . Poisson regression analysis of factors associated with the number of buprenorphine prescriptions across 6 months.

	**Unadjusted model**			**Adjusted model**		
**Variable**	**IRR**	**95% CI**	***p***-value	**IRR**	**95% CI**	***p***-value
**Sex**						
Male	Ref.	—-	—-	Ref.	—-	—-
Female	1.08	0.99–1.18	0.09	1.09	0.99–1.20	0.08
**Age (years) on Jan. 1, 2010**						
18–25						
36–34	1.05	0.92–1.20	0.46	1.03	0.90–1.18	0.62
35–49	1.03	0.92–1.16	0.63	1.04	0.92–1.17	0.55
50+	1.00	0.87–1.16	0.96	1.00	0.86–1.15	0.98
**Race/ethnicity**						
White, non-Hispanic	Ref.	—-	—-			
Black, non-Hispanic	0.97	0.88–1.08	0.60	0.99	0.90–1.10	0.86
Other, Hispanic	1.19	0.97–1.47	0.09	1.21	0.99–1.49	0.07
**Type of CNCP condition**						
Any chronic pain	**1.13**	**1.02–1.25**	**<0.05**	**1.20**	**1.07–1.34**	**<0.01**
General chronic pain	**1.15**	**1.02–1.30**	**<0.05**	—-	—-	—-
Back pain	1.08	0.96–1.23	0.21	—-	—-	—-
Migraine/headache	1.03	0.74–1.43	0.86	—-	—-	—-
Osteoarthrosis	1.14	0.89–1.47	0.29	—-	—-	—-
Musculoskeletal pain	**1.21**	**1.02–1.44**	**<0.05**	—-	—-	—-
**Comorbid psychiatric disorder diagnoses**						
Any psychiatric disorder	0.99	0.89–1.10	0.87	0.95	0.84–1.07	0.41
Mood disorder (any)	1.03	0.91–1.16	0.62	—-	—-	—-
Anxiety disorder (any)	1.05	0.93–1.19	0.44	—-	—-	—-
Other psychiatric disorder	1.01	0.87–1.17	0.90	—-	—-	—-
**Comorbid substance use disorders**						
Any substance use disorder	0.94	0.84–1.05	0.25	0.90	0.80–1.02	0.09
Tobacco use disorder	0.98	0.85–1.12	0.73	—-	—-	—-
Alcohol use disorder	1.04	0.87–1.24	0.68	—-	—-	—-
Stimulant use disorder	0.96	0.81–1.15	0.69	—-	—-	—-
Other drug use disorder	0.90	0.77–1.04	0.16	—-	—-	—-
**Daily dose of last buprenorphine prescription**						
Low (<16 mg)	Ref.	—-	—-	Ref.	—-	—-
Medium (16–23 mg)	**1.14**	**1.03–1.26**	**<0.05**	**1.14**	**1.02–1.26**	**<0.05**
High (≥24 mg)	1.12	0.97–1.28	0.12	1.12	0.97–1.28	0.12
Missing	**0.76**	**0.59–0.96**	**<0.05**	**0.74**	**0.58–0.95**	**<0.05**

Note: Statistically significant (*p* < 0.05) results are bolded. *The adjusted model was controlled for the variables shown in column including sex, age, race/ethnicity, any chronic pain, any comorbid psychiatric disorder, any comorbid substance use disorder, and daily buprenorphine dose. ^§^Other mental health disorders included psychotic, sleep, adjustment, personality, attention-deficit/impulse/conduct, somatoform, or eating disorders. ^¶^Other drug use disorders included cannabis, sedative/hypnotic/anxiolytic, hallucinogen, inhalant, or other/unspecified drug use disorder. IRR: Incidence rate ratio; CI: Confidence interval; CNCP: chronic non-cancer pain.

## 4 Discussion

In this study of EHR data from a large healthcare system, we examined the association of co-occurring CNCP and 6-month buprenorphine treatment retention among individuals with OUD. Among this sample, those with co-occurring CNCP were of relatively older age and had more psychiatric and substance use disorder (SUD) comorbidities than those without co-occurring CNCP. After controlling for sociodemographic characteristics, psychiatric and SUD comorbidities, and daily buprenorphine dose, we found that the baseline presence of any CNCP condition or specific types of chronic pain conditions was not associated with time to buprenorphine treatment discontinuation. However, the baseline presence of any CNCP condition was associated with a higher number of buprenorphine prescriptions across 6 months. Furthermore, we found that sociodemographic and other clinical factors (i.e., psychiatric and substance use disorder comorbidities, daily buprenorphine dose) in our analysis did not moderate the association of CNCP status and time to buprenorphine treatment discontinuation or number of buprenorphine prescriptions. Together, these data add to the existing literature by leveraging EHR data to generate evidence within a real-world setting.

Given that the time to treatment discontinuation was not different as a function of CNCP status in our sample, the results from this study support the use of buprenorphine as a viable treatment option in patients with OUD and co-occurring CNCP with regard to treatment retention outcomes. Previous research also shows that buprenorphine can be effective at reducing pain in individuals with OUD and CNCP (Roux et al., 2013), which further supports its utility in this patient subpopulation. Moreover, buprenorphine can be prescribed by waivered providers in office-based settings (e.g., primary care), which may make it a relatively more accessible treatment option for some patients than methadone maintenance treatment (Edelman et al., 2018; Volkow et al., 2014). Nevertheless, it should be noted that buprenorphine retention rates were low across both groups, which is consistent with other studies among individuals with OUD and underscores the need for more effective treatment strategies that address barriers to treatment retention in general (Samples et al., 2018). Low overall retention rates in our sample may have been particularly driven by difficulties during the induction phase of treatment given that a large proportion met treatment discontinuation criteria within the first 10 days of treatment. These findings further highlight the induction phase as a critical treatment period in which enhanced support may be needed to facilitate favorable outcomes (Jacobs et al., 2015).

While treatment discontinuation did not differ by CNCP status in our sample, our finding that co-occurring CNCP was associated with a greater number of buprenorphine prescriptions across 6 months suggests treatment differences that may have clinical implications and should be further investigated. It is possible that this finding reflects a greater likelihood of individuals with comorbid CNCP to return to buprenorphine treatment after initially discontinuing treatment. This finding may also reflect potentially different buprenorphine prescribing patterns for patients with CNCP in our sample (e.g., fewer/greater quantity prescribed) or unmeasured confounders in our dataset, such as intermittently receiving treatment outside the healthcare system. Additional research is needed to confirm these findings and to explore potential underlying mechanisms. This information can be valuable to clinicians for informing targeted treatment management strategies.

Our finding that no patient-level factors moderated the association of CNCP and buprenorphine treatment retention was somewhat surprising. That is, previous studies suggest that sociodemographic and clinical characteristics can influence disparities in the quality of pain treatment or the functional interference of pain that could thereby pose a risk for early OUD treatment discontinuation. For example, studies have shown that racial/ethnic minorities and females are less likely to receive prescriptions for pain medication and lower doses of medication despite reporting more frequent and severe pain compared to males and non-Hispanic white patients, respectively (Haq et al., 2021; Hirsh et al., 2015; Hurley and Adams, 2008; Kaplovitch et al., 2015; Kennel et al., 2019; Wisniewski et al., 2008). Additionally, previous research among individuals reporting past-month drug use and chronic pain has shown an association between mental health comorbidity and greater pain-related functional interference (Voon et al., 2021). Research also suggests that the daily dose of buprenorphine could be a key factor influencing treatment outcomes in patients with co-occurring CNCP. A positive association between buprenorphine dose and pain reduction has been reported in patients with OUD and co-occurring CNCP up to 16 mg (Roux et al., 2013), suggesting relatively lower doses could pose a risk for treatment discontinuation as a result of reduced pain management. While we did not see a moderating effect of these factors on the association of CNCP and buprenorphine retention, it is possible that our findings reflect unmeasured confounders that may influence the effect of CNCP on retention such as baseline severity of pain, pain interference, change in pain over time, and provider treatment bias. A better understanding of these relationships will be important for identifying potential pain management needs among patients receiving OUD treatment that may also be a barrier to optimal treatment retention.

Our findings also have clinical implications for the use of a patient's CNCP status as a prognostic indicator during buprenorphine maintenance treatment. The results from this study add to the results from other studies also showing no association between CNCP status and buprenorphine treatment retention (Bounes et al., 2013; Fox et al., 2012; Peck et al., 2021). Thus, these results suggest that the mere presence of CNCP among individuals with OUD at treatment initiation may hold little predictive validity for treatment retention outcomes. Instead, research suggests the extent to which CNCP affects treatment outcomes may depend more on certain temporal aspects of CNCP. For instance, two studies showed that greater levels of pain and fluctuations in pain over time predicted increased odds of opioid use during buprenorphine treatment (Worley et al., 2015, 2017). Additional research is needed to determine whether this effect also extends to other outcomes such as retention in treatment, which could be useful for better understanding CNCP as a prognostic risk factor and informing strategies for maximizing the benefit of buprenorphine treatment.

There are limitations to this study that should be taken into consideration when interpreting the findings. First, our findings are correlational in nature, which preclude causal inference for significant effects. Our findings may also be limited by selection bias given the small proportion of patients with OUD in our sample that received treatment. This pattern, however, is consistent with national data on OUD treatment utilization (Wu et al., 2016). Additionally, our study relied on diagnosis codes within the EHR dataset for identifying patients with CNCP and other conditions. However, as with any retrospective study based on administrative data collected as part of routine care, the accuracy of diagnosis codes to identify patients in the sample may be influenced by several factors including provider specialty and training, patient demographics, insurance coverage (billable diagnoses), and/or variation in clinical screening/assessment practices. The use of diagnosis codes to identify patients with chronic pain in particular have been shown to have high specificity, although sensitivity is likely lower (Tian et al., 2013). Also inherent to EHR and claims-based analysis in general, it is unknown whether prescribed buprenorphine was filled at a pharmacy and consumed by the patient or diverted. Furthermore, our findings may have been biased because EHR data does not capture treatment received outside of the healthcare system from which the EHR data was collected. Thus, there is the possibility that buprenorphine treatment discontinuation was overestimated in our sample as a result of patients transferring treatment outside of the Duke University healthcare system. Finally, it should be acknowledged that the generalization of findings to other states or regions may be limited.

Notwithstanding these limitations, the use of EHR data for this study extends previous research on the association of CNCP and buprenorphine retention. Previous research has been conducted within the context of specific patient subpopulations including one study of Medicaid beneficiaries, and two other studies among a relatively small cohort of patients with comorbid HIV and without psychiatric comorbidities, respectively (Fox et al., 2012; Peck et al., 2021; Samples et al., 2018), thereby limiting the generalization of findings to broader samples of patients. Thus, the use of EHR data for this study afforded the opportunity to address some of the limitations of previous studies by providing a diverse sample and one that is reflective of patient/provider behavior in a real-world setting.

## 5 Conclusion

This study further adds to the emerging knowledge base concerning the impact of chronic non-cancer pain on buprenorphine treatment outcomes for individuals with OUD. In this sample, the presence of CNCP at baseline was not associated with time to buprenorphine treatment discontinuation but was associated with a greater number of buprenorphine prescriptions received across 6 months, while controlling for confounding factors. We also found that sociodemographic and clinical factors (i.e., psychiatric comorbidities, daily buprenorphine dose) did not moderate the association of CNCP status and time to buprenorphine treatment discontinuation. These findings suggest that buprenorphine is a viable treatment option for patients with co-occurring CNCP and OUD, while the presence of co-occurring CNCP alone cannot be reliably associated with treatment retention outcomes. Nonetheless, the presence of co-occurring chronic pain among patients with OUD can pose treatment challenges and clinical ambiguity to providers (Berg et al., 2009). Thus, treatment providers need to be aware of the patient's chronic pain and attend to related outcomes. The use of adjunct pain therapies such as NSAID's, selected antidepressants, or non-pharmacologic therapies while treating with buprenorphine may also be a useful strategy (Koller et al., 2019). There is also a need for additional research of factors that may obscure the association between CNCP and buprenorphine treatment outcomes to inform strategies at optimizing treatment among this prevalent patient subgroup.

## CRediT authorship contribution statement

**William S. John:** Funding acquisition, Conceptualization, Data curation, Methodology, Formal analysis, Writing – original draft. **Paolo Mannelli:** Conceptualization, Writing – original draft. **Rick H. Hoyle:** Methodology, Writing – original draft. **Lawrence Greenblatt:** Writing – original draft. **Li-Tzy Wu:** Funding acquisition, Conceptualization, Methodology, Writing – original draft.

## Declaration of Competing Interest

Li-Tzy Wu also has received research funding from Patient-Centered Outcomes Research Institute and Centers for Disease Control and Prevention. William S. John also has received research funding from Patient-Centered Outcomes Research Institute. Paolo Mannelli has received research support from Alkermes Inc. and Orexo, and is a consultant for Alkermes, Intracellular Therapies, Atai Life Sciences, and Guidepoint Global. The other authors have no conflicts of interest to disclose.

## Appendix Supplementary materials

Image, application 1
