# Supplementary material for: Association of chronic non-cancer pain status and buprenorphine treatment retention among individuals with opioid use disorder: Results from electronic health record data
Source: Drug Alcohol Depend Rep. 2022 Mar 26;3:100048. doi: 10.1016/j.dadr.2022.100048 (PMC9948869; doi:10.1016/j.dadr.2022.100048)
Supplement: Supplementary file 1 [file mmc1.docx]

**Table S1.** ICD-10-CM and ICD-9-CM codes utilized for study variables.

| **Variable** | **Codes** |
| --- | --- |
| **Chronic non-cancer pain** | B02.22, F45.4, G43, G44, G50, G54.0, G54.6, G56, G57.7, G58.7, G58.9, G89.0, G89.2, G89.4, G90.5, M02.0, M02.9, M05, M06, M07, M08, M1A, M10, M11, M12.0, M12.1, M12.5, M12.8, M12.9, M14, M15, M16, M17, M18, M19, M20.1, M21.61, M21.62, M22.4, M23, M24.0, M25.5, M25.7, M31.5, M32, M33, M34, M35, M36, M43.2, M43.8X9, M45, M46.0, M46.1, M46.4, M46.5, M46.8, M46.9, M47, M48.0, M48.1, M48.2, M48.3, M48.8, M48.9, M49.8, M50, M51, M53.0, M53.1, M53.2, M53.3, M53.8, M53.9, M54, M60.8, M60.9, M65, M67.3, M67.4, M70.0, M70.1, M70.2, M70.3, M70.4, M70.5, M70.6, M70.7, M71.1, M71.3, M71.5, M72.8, M72.9, M75, M76, M77, M79.0, M79.1, M79.2, M79.6, M79.7, M79.A, M79.89, M79.9, M96.1, S13, S23, S33, S83, 274, 307.8, 337.2, 338, 339, 346, 350, 353.0, 353.6, 354, 355.71, 355.9, 710, 712, 713, 714, 715, 716, 717, 718.0, 718.1, 719.4, 720, 721, 722, 723, 724, 725, 726, 727.0, 727.1, 727.2, 727.3, 727.4, 729.0, 729.1, 729.2, 729.4, 729.5, 729.7, 729.9, 780  *Excluded*: G89.3, 338.3 Neoplasm Related Chronic Pain |
| **SUD diagnoses** |  |
| Opioid | F11.10, F11.120, F11.121, F11.122, F11.129, F11.14, F11.151, F11.159, F11.188, F11.19, F11.2, F11.20, F11.220, F11.221, F11.222, F11.229, F11.23, F11.24, F11.250, F11.259, F11.281, F11.282, F11.288, F11.29, F11.90, F11.920, F11.921, F11.929, F11.93, F11.94, F11.951, F11.959, F11.981, F11.982, F11.988, F11.99, 304.00, 304.01, 304.02, 304.70, 304.71, 304.72, 305.50, 305.51, 305.52 |
| Tobacco | F17.200, F17.203, F17.208, F17.209, F17.210, F17.213, F17.218, F17.219, F17.220, F17.223, F17.228, F17.229, F17.290, F17.293, F17.298, F17.299, 305.1 |
| Alcohol | F10.10, F10.120, F10.121, F10.129, F10.14, F10.150, F10.151, F10.159, F10.180, F10.181, F10.188, F10.19, F10.20, F10.220, F10.221, F10.229, F10.230, F10.231, F10.232, F10.239, F10.24, F10.250, F10.251, F10.259, F10.26, F10.27, F10.280, F10.282, F10.288, F10.29, F10.920, F10.921, F10.929, F10.94, F10.950, F10.951, F10.959, F10.96, F10.97, F10.980, F10.982, F10.988, F10.99, 291.0, 291, 291.1, 291.2, 291.3, 291.4, 291.5, 291.8, 291.81, 291.82, 291.89, 291.9, 303, 303.00, 303.01, 303.02, 303.9, 303.90, 303.91, 303.92, 305.0, 305.00, 305.01, 305.02 |
| Stimulant | F14.10, F14.120, F14.121, F14.122, F14.129, F14.14, F14.150, F14.151, F14.159, F14.180, F14.188, F14.19, F14.20, F14.220, F14.221, F14.222, F14.229, F14.23, F14.24, F14.250, F14.251, F14.259, F14.280, F14.288, F14.29, F14.90, F14.920, F14.921, F14.922, F14.929, F14.94, F14.950, F14.951, F14.959, F14.980, F14.982, F14.988, F14.99, F15.10, F15.120, F15.121, F15.129, F15.14, F15.150, F15.151, F15.159, F15.180, F15.182, F15.188, F15.19, F15.20, F15.229, F15.23, F15.24, F15.251, F15.259, F15.90, F15.920, F15.921, F15.922, F15.929, F15.93, F15.94, F15.950, F15.951, F15.959, F15.980, F15.982, F15.988, F15.99, 304.2, 304.20, 304.21, 304.22, 304.40, 304.41, 304.42, 305.6, 305.60, 305.61, 305.62, 305.70, 305.71, 305.72 |
| Other drug | *Cannabis:* F12.10, F12.120, F12.121, F12.122, F12.129, F12.150, F12.151, F12.159, F12.180, F12.188, F12.19, F12.20, F12.221, F12.222, F12.229, F12.23, F12.250, F12.251, F12.259, F12.288, F12.29, F12.90, F12.920, F12.921, F12.922, F12.929, F12.93, F12.950, F12.951, F12.959, F12.980, F12.988, F12.99, 304.3, 304.30, 304.31, 304.32, 305.2, 305.20, 305.21, 305.22  *Sedative/hypnotic/anxiolytic:* F13.10, F13.120, F13.121, F13.129, F13.14, F13.159, F13.180, F13.188, F13.19, F13.20, F13.229, F13.230, F13.231, F13.232, F13.239, F13.24, F13.280, F13.288, F13.90, F13.920, F13.921, F13.929, F13.939, F13.94, F13.950, F13.951, F13.959, F13.97, F13.980, F13.982, F13.99, 304.10, 304.11, 304.12, 305.40, 305.41, 305.42  *Hallucinogen:* F16.10, F16.129, F16.14, F16.150, F16.151, F16.159, F16.183, F16.19, F16.20, F16.283, F16.90, F16.920, F16.921, F16.929, F16.94, F16.950, F16.951, F16.959, F16.980, F16.983, F16.988, F16.99, 304.50, 304.51, 304.52, 305.30, 305.31, 305.32  *Inhalant:* F18.10, F18.129, F18.14, F18.159, F18.20, F18.220, F18.229, F18.90, F18.929, F18.988  *Other/unspecified:* F19.10, F19.120, F19.121, F19.129, F19.14, F19.151, F19.159, F19.180, F19.188, F19.19, F19.20, F19.220, F19.221, F19.229, F19.230, F19.231, F19.232, F19.239, F19.24, F19.250, F19.251, F19.259, F19.280, F19.288, F19.29, F19.90, F19.920, F19.921, F19.922, F19.929, F19.930, F19.931, F19.939, F19.94, F19.950, F19.951, F19.959, F19.96, F19.97, F19.980, F19.981, F19.982, F19.988, F19.99, F55.0, F55.1, F55.2, F55.3, F55.8, 304.60, 304.61, 304.62, 304.9, 304.90, 304.91, 304.92, 305.80, 305.81, 305.9, 305.90, 305.91, 305.92 |
| **Psychiatric disorder diagnoses** |  |
| Depressive disorder | F32.0, F32.1, F32.2, F32.3, F32.4, F32.5, F32.89, F32.9, F33.0, F33.1, F33.2, F33.3, F33.40, F33.41, F33.42, F33.8, F33.9, F34.1, 296.20, 296.2, 296.21, 296.22, 296.23, 296.24, 296.25, 296.26, 296.30, 296.3, 296.31, 296.32, 296.33, 296.34, 296.35, 296.36, 296.82, 300.4, 311 |
| Bipolar disorder | F30.10, F30.11, F30.12, F30.2, F30.4, F30.8, F30.9, F31.0, F31.10, F31.11, F31.12, F31.13, F31.2, F31.30, F31.31, F31.32, F31.4, F31.5, F31.60, F31.61, F31.62, F31.63, F31.64, F31.70, F31.71, F31.72, F31.73, F31.74, F31.75, F31.76, F31.77, F31.78, F31.81, F31.89, F31.9, 296.00, 296.0, 296.01, 296.02, 296.03, 296.04, 296.05, 296.06, 296.10, 296.11, 296.12, 296.13, 296.14, 296.15, 296.16, 296.40, 296.4, 296.41, 296.42, 296.43, 296.44, 296.45, 296.46, 296.50, 296.5, 296.51, 296.52, 296.53, 296.54, 296.55, 296.56, 296.60, 296.61, 296.62, 296.63, 296.64, 296.65, 296.66, 296.7, 296.80, 296.8, 296.81, 296.89 |
| Mood disorder (any) | F06.30, F06.31, F06.32, F06.33, F06.34, F10.14, F10.24, F10.94, F11.14, F11.24, F11.94, F13.14, F13.24, F13.94, F14.14, F14.24, F14.94, F15.14, F15.24, F15.94, F16.14, F16.94, F18.14, F19.14, F19.24, F19.94, F30.10, F30.11, F30.12, F30.2, F30.4, F30.8, F30.9, F31.0, F31.10, F31.11, F31.12, F31.13, F31.2, F31.30, F31.31, F31.32, F31.4, F31.5, F31.60, F31.61, F31.62, F31.63, F31.64, F31.70, F31.71, F31.72, F31.73, F31.74, F31.75, F31.76, F31.77, F31.78, F31.81, F31.89, F31.9, F32.0, F32.1, F32.2, F32.3, F32.4, F32.5, F32.89, F32.9, F33.0, F33.1, F33.2, F33.3, F33.40, F33.41, F33.42, F33.8, F33.9, F34.0, F34.1, F34.81, F34.89, F34.9, F39, 292.84, 293.83, 296.00, 296.0, 296.01, 296.02, 296.03, 296.04, 296.05, 296.06, 296.10, 296.11, 296.12, 296.13, 296.14, 296.15, 296.16, 296.20, 296.2, 296.21, 296.22, 296.23, 296.24, 296.25, 296.26, 296.30, 296.3, 296.31, 296.32, 296.33, 296.34, 296.35, 296.36, 296.40, 296.4, 296.41, 296.42, 296.43, 296.44, 296.45, 296.46, 296.50, 296.5, 296.51, 296.52, 296.53, 296.54, 296.55, 296.56, 296.60, 296.61, 296.62, 296.63, 296.64, 296.65, 296.66, 296.7, 296.80, 296.8, 296.81, 296.82, 296.89, 296.90, 296.9, 296.99, 300.4, 301.13, 311 |
| Anxiety disorder | F06.4, F40.00, F40.01, F40.02, F40.10, F40.11, F40.210, F40.218, F40.220, F40.228, F40.231, F40.232, F40.233, F40.240, F40.241, F40.242, F40.243, F40.248, F40.298, F40.8, F40.9, F41.0, F41.1, F41.3, F41.8, F41.9, F42, F42.2, F42.3, F42.4, F42.8, F42.9, F43.0, F43.10, F43.11, F43.12, F44.9, R45.5, R45.6, R45.7, F48.8, F48.9, F93.8, F99, 293.84, 300.00, 300.01, 300.02, 300.09, 300.10, 300.20, 300.21, 300.22, 300.23, 300.29, 300.3, 300.5, 300.9, 308.0, 308.1, 308.2, 308.3, 308.4, 308.9, 309.21, 309.81, 313.0, 313.1, 313.21, 313.22, 313.3, 313.82, 313.83 |
| Other psychiatric disorder | *Psychotic disorder:* F06.0, F06.2, F20.0, F20.1, F20.2, F20.3, F20.5, F20.81, F20.89, F20.9, F21, F22, F23, F24, F25.0, F25.1, F25.8, F25.9, F28, F29, F44.89, 291.3, 291.5, 292.11, 292.12, 293.81, 293.82, 295.00, 295.01, 295.02, 295.03, 295.04, 295.05, 295.10, 295.11, 295.12, 295.13, 295.14, 295.15, 295.20, 295.21, 295.22, 295.23, 295.24, 295.25, 295.3, 295.30, 295.31, 295.32, 295.33, 295.34, 295.35, 295.40, 295.41, 295.42, 295.43, 295.44, 295.45, 295.50, 295.51, 295.52, 295.53, 295.54, 295.60, 295.61, 295.62, 295.63, 295.64, 295.65, 295.70, 295.71, 295.72, 295.73, 295.74, 295.75, 295.80, 295.81, 295.82, 295.83, 295.84, 295.85, 295.90, 295.9, 295.91, 295.92, 295.93, 295.94, 295.95, 297.0, 297.1, 297.2, 297.3, 297.8, 297.9, 298.0, 298.1, 298.2, 298.3, 298.4, 298.8, 298.9  *Sleep disorder:* 307.40, 307.41, 307.42, 307.43, 307.44, 307.45, 307.46, 307.47, 307.48, 307.49, 327.21, 327.23, 327.24, 327.25, 327.26, 327.42, 333.94, 347, 347.00, 347.01, 347.10, 347.11, 780.5, 780.50, 780.51, 780.52, 780.53, 780.54, 780.55, 780.56, 780.57, 780.58, 780.59, 780.6, 76.04, F51.01, F51.04, F51.09, F51.11, F51.19, F51.3, F51.4, F51.5, F51.8, F51.9, G25.81, G47.00, G47.09, G47.10, G47.11, G47.12, G47.13, G47.19, G47.20, G47.21, G47.22, G47.23, G47.24, G47.25, G47.26, G47.27, G47.29, G47.30, G47.31, G47.33, G47.34, G47.35, G47.36, G47.37, G47.39, G47.411, G47.419, G47.421, G47.429, G47.50, G47.51, G47.52, G47.53, G47.59, G47.8, G47.9  *Adjustment disorders*: F43.20, F43.21, F43.22, F43.23, F43.24, F43.25, F43.29, 309.0, 309.1, 309.22, 309.23, 309.24, 309.28, 309.29, 309.3, 309.4, 309.82, 309.83, 309.89, 309.9  *Personality disorders*: F60.0, F60.1, F60.2, F60.3, F60.4, F60.5, F60.6, F60.7, F60.81, F60.89, F60.9, F68.8, F69, 301.0, 301, 301.10, 301.11, 301.12, 301.20, 301.21, 301.22, 301.3, 301.4, 301.50, 301.59, 301.6, 301.7, 301.81, 301.82, 301.83, 301.84, 301.89, 301.9  *Attention-deficit/impulse/conduct disorders*: F63.0, F63.2, F63.3, F63.81, F63.89, F63.9, F90.0, F90.1, F90.2, F90.8, F90.9, F91.0, F91.1, F91.2, F91.3, F91.8, F91.9, 312.00, 312.01, 312.02, 312.03, 312.10, 312.11, 312.12, 312.13, 312.20, 312.21, 312.23, 312.30, 312.3, 312.31, 312.32, 312.33, 312.34, 312.35, 312.39, 312.4, 312.8, 312.81, 312.82, 312.89, 312.9, 313.81, 313.82, 313.89, 314.00, 314.0, 314.01, 314.1, 314.2, 314.8, 314.9  *Somatoform disorders*: F44.4, F44.5, F44.6, F44.7, F45.0, F45.1, F45.20, F45.21, F45.22, F45.8, F45.9, F54, F68.10, F68.11, F68.12, F68.13, 300.11, 300.16, 300.19, 300.7, 300.81, 300.82, 300.89, 301.51, 306.0, 306.1, 306.2, 306.3, 306.4, 306.50, 306.51, 306.52, 306.53, 306.59, 306.6, 306.7, 306.8, 306.9, 316  *Eating disorders*: F50.00, F50.01, F50.02, F50.2, F50.8, F50.81, F50.82, F50.89, F50.9, F98.21, F98.29, F98.3, 307.1, 307.5, 307.50, 307.51, 307.52, 307.53, 307.54, 307.59 |
